# Bisegmental posterior stabilisation of thoracolumbar fractures with polyaxial pedicle screws: Does additional balloon kyphoplasty retain vertebral height?

**DOI:** 10.1371/journal.pone.0233240

**Published:** 2020-05-18

**Authors:** Julia Starlinger, Greta Lorenz, Alexandra Fochtmann-Frana, Kambiz Sarahrudi

**Affiliations:** 1 Department for Orthopedics, Mayo Clinic, Rochester, MN, United States of America; 2 Department for Orthopedics and Trauma Surgery, Medical University Vienna, Vienna, Austria; 3 Trauma Center Meidling, Wien, Austria; 4 Department for Plastic Surgery, Medical University Vienna, Vienna, Austria; 5 Department for Trauma Surgery, Landesklinikum Wiener Neustadt, Wiener Neustadt, Austria; Assiut University Faculty of Medicine, EGYPT

## Abstract

We retrospectively evaluated single-level compression fractures (T12-L3) scheduled for a short-segment POS (posterior-only stabilization) using polyaxial screws. Patients averaged 55.7 years (range, 19–65). Patients received either POS or, concomitantly, BK (balloon kyphoplasty) of the fractured vertebrae as well. Primary endpoint was the radiological outcome at the last radiographic follow-up prior to implant removal. POS together with BK of the fractured vertebrae resulted in a significant improvement of the local kyphosis angle and vertebral body compression rates immediately post-OP. During the further course of FU, a considerable loss of correction was observed post-OP in both groups. (Local KA: pre-OP/ post-OP/ FU: 12.6±4.8/ 3.35±4.8/ 11.6±6.0; anterior vertebral body compression%: pre-OP/post-OP/ FU: 71.94±12.3/ 94.78±19.95/ 78.17±14.74). VAS was significantly improved from 7.2±1.3 pre-OP to 2.7±1.3 (P<0.001) at FU. We found a significant restoration of the vertebral body height by BK. Nevertheless, follow-up revealed a noticeable loss of reduction. Given the fact that BK used together with polyaxial screws did not maintain intra-operative reduction, our data do not support this additional maneuver when used together with bi-segmental polyaxial pedicle screw fixation.

## Introduction

Type A compression fractures of the TL (thoracolumbar) spine are frequent but the fracture morphology is heterogeneous, and so are recent classifications and consecutive treatment recommendations [1–3]. Moreover, treatment recommendations for type A fractures depend on various factors such as patients bone quality, co-morbidities as well as surgeons’ preference and experience. With respect to fracture morphology as well as pre-existing spinal deformity, the load-bearing capacity of the anterior column might be altered, which ultimately puts the patients sagittal balance at risk.

Stand-alone BK (balloon kyphoplasty) was found to be a feasible therapy of vertebral compression fractures, allowing for satisfactory intra-operative anterior column restoration. Yet various studies have revealed its limited capacity of withstanding axial load forces, resulting in secondary loss of correction [4]. Since then BK alone is only used for a very select group of patients.

POS (posterior-only stabilization) is a well-established and relatively safe procedure for treating vertebral compression fractures, especially since MIS (minimal invasive surgery) minimized approach-related morbidity [5]. While studies comparing POS and BK revealed superior results for POS [6], loss of reduction remains a relevant limitation observed in both procedures. In particular, it is well known that POS of burst fractures without sufficient support of the anterior column leads to loss of reduction after implant removal [7–9].

In an effort to establish a technique that assures long-term reduction, the use of BK as additional augmentation of the fractured vertebrae together with posterior instrumentation was promoted.

Satisfactory anterior column support and a concomitant reduction of additional anterior procedures have been reported upon using this approach [10, 11]. However, several limitations in that respect are inherent in existing reports. The fact that the number of spinal levels included in the instrumentation as well as fracture types and levels are heterogeneous [12–14], and that sample sizes are small and lack control groups represents a relevant disadvantage in these studies [11, 15, 16].

But burst fractures are frequent, which makes the lack of consensus even more clinically relevant. Especially in young, non-osteoporotic patients it is of utmost importance to reconstruct the pre-injury anatomy as well as the individual pre-injury sagittal profile, as recently emphasized by the Spine Section of the German Society for Orthopedics and Trauma [17].

Particularly in those young (<65a), non-osteoporotic patients, one would expect an adequate bone healing response. Therefore, we speculated that a combination of POS and BK, might maintain intra-operative reduction. We hypothesize that the use of additional BK increases the support of the anterior column and would therefore overcome the biomechanical limitations of polyaxial screws in the context of fracture fixation. This could ultimately combine the advantages of polyaxial screws causing as little surgical trauma as possible together with adequate restoration of the anterior column by the use of additional BK.

We therefore reached out to assess if BK, using calcium phosphate cement, together with bisegmental POS, using polyaxial screws, could compensate for the weaknesses of POS alone and provide for superior results at follow-up in regards to radiographical as well as clinical outcomes.

## Materials and methods

We performed a retrospective cohort study including all consecutive patients who sustained a single-level vertebral compression fracture of the TL spine (T12—L3) (Type A fractures, according to the new AOSpine Classification [2]) and were scheduled for a short-segment posterior-only pedicle screw instrumentation (one level cephalad and caudad to the fractured vertebra). In order to ensure a homogenous collective, we imposed strict inclusion and exclusion criteria that are listed in Table 1.

**Table 1:** Inclusion and exclusion criteria.

Inclusion criteria	Exclusion criteria
Patients between 18 and 65 years of age	Absence of a traumatic event
Fractures of vertebrae Th12- L3	Long-segment fixation
Single level vertebral compression fracture (A1, A2 and A3)	Use of intermediate screws
Posterior bi-segmental stabilization within 14 days after trauma	Posterior ligamentous complex injuries
	Osteoporosis
	Cement augmented screws due to osteoporosis
	Patients who were initially considered for a 360° fusion
	Revision surgery
	Preoperative neurologic deficit
	History of spinal infection
	History of malignant neoplasm
	Metal allergies
	Any contraindications to CT or MRI
	Pre-existing serious spinal column deformity

Doing so, a total of 42 consecutive patients were included in the study. Table 2 illustrates the respective fracture morphology and distribution between the study groups.

**Table 2:** Fracture pattern amongst groups.

Control Group	Fractured Vertebra	Study Group
3,3,3,3,3;	**Th12**	2,3,3,3;
3,3;	**L1**	1,2,2,3,3,3,3,3,3,3,3,3;
1,3;	**L2**	1,1,2,2,3,3,3,3,3,3,3;
3,3,3;	**L3**	2,3;

Stem and leaf plot illustrates the distribution of fracture pattern between the two groups according to „Type A”of the AO fracture classification [2].

All patients were treated at the Medical University of Vienna, Department of Orthopedics and Trauma Surgery, which is a level-one trauma center, from January 2014 to March 2017. Patients were divided into two groups based on their surgical procedure. In particular, 12 patients received posterior stabilization alone (6 males, 6 females). They will be referred to as the control group. A total of 30 patients (22 males, 8 females) underwent posterior stabilization and additionally received bipedicular percutaneous BK of the fractured vertebrae. They therefore comprised the study group.

The study was approved by the Institutional Review Board (IRB) (Ethics Committee of the Medical University of Vienna 1011/2020) and conducted in accordance to the declaration of Helsinki. Due to the retrospective nature of the cohort study no informed consent was required by the IRB. After radiographic measurements and prior to statistical work-up patients records were anonymized.

### Surgical procedure

All patients underwent bisegmental POS in prone position under general anesthesia. Surgery was performed by or under the supervision of an experienced spine surgeon. Using a typical minimal invasive approach, four polyaxial pedicle screws (Longitude, Medtronic, Memphis, TN, USA) were implanted under fluoroscopic guidance. Indirect reduction of the fractured vertebra via ligamentotaxis was performed.

In the study group direct reduction was performed by percutaneous bipedicular BK of the fractured vertebra. The balloon (Kyphon®, Medtronic, Memphis, TN, USA) was slowly inflated and endplate reduction performed under X-ray control. The balloon was then removed and bone cement was applied corresponding to the volume of the vertebra as well as to the degree of destruction. The volume of injected calcium phosphate cement averaged 5,5 ml (range, 3–6 ml) per vertebra. Decision for the use of additional kyphoplasty was based on the surgeon’s preference.

### Postoperative procedure

Patients were fully mobilized on the first day after surgery under the close supervision of physiotherapists, according to a standardized rehabilitation program.

### Assessment of the patients

Preoperative radiographic assessment included plain X-rays in supine position (a-p and lateral view) as well as a CT scan. Prior to surgery all patients underwent an MRI scan to evaluate PLC (posterior ligamentous complex) injuries and detect occult fractures of adjacent vertebrae. All postoperative plain X-rays evaluated position of implants, fracture reduction, and restoration of lumbar lordosis.

Three independent observers evaluated all CT and MRI scans for accurate fracture classification according to the AOSpine Classification [2] as well as plain radiographs to assess kyphotic deformity using Cobb measurement. This included the monosegmental Cobb angle, measured between superior and inferior endplate of the fractured vertebrae, and measuring the local kyphosis angle (KA)(°) [1, 18]. Further, the bisegmental Cobb angle, measured between the superior endplate of the intact vertebra cephalad to the fracture and between the inferior endplate of the vertebra caudad to the fracture.

We measured anterior VB height (AVH) and posterior VB height (PVH) in lateral spine radiographs or mid-sagittal CT images of the fractured and adjacent VB.

**Fig 1:**
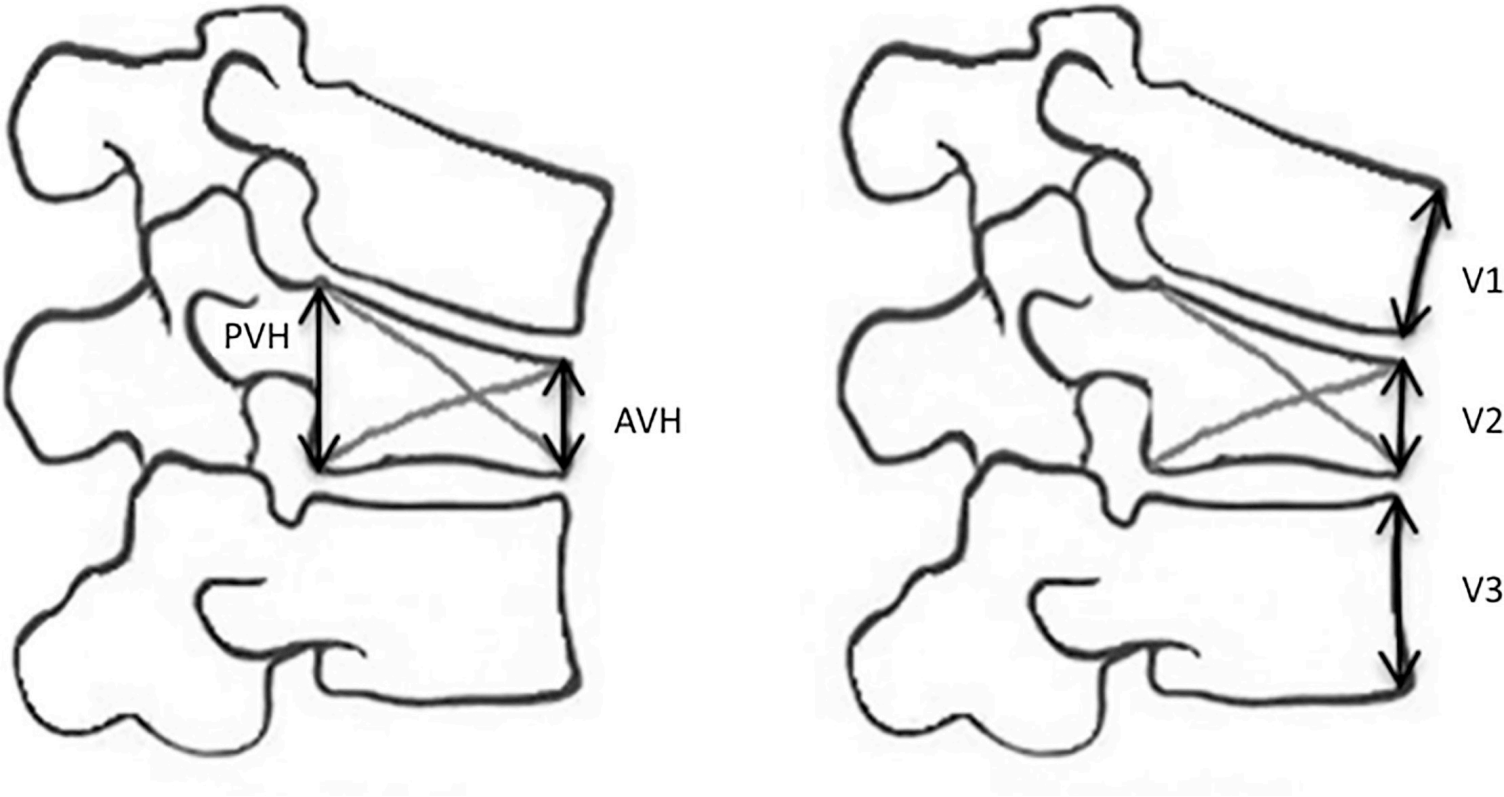
Radiographic measurements. Measurement of VBCR (VB compression rate) = AVH/PVH, AVH anterior vertebral height, PVH posterior vertebral height; Measurement of AVBC% (Anterior VB compression percentage) = V2/[(V1+V3)/2]x100% [19].

Two methods for calculating VBHL (VB height loss) were used: The VBCR (VB compression rate), i.e., the ratio of AVH to PVH with the formula VBCR = AVH/PVH. The VBCR is recommended to assess the structural integrity of the fractured VB, specifically, that of the anterior and middle columns of the injured vertebra (Fig 1, from [19]).

We further calculated the AVBC % (Anterior VB Compression Percentage), as recommended by the Spine Trauma Study Group to assess VBHL. The AVBC % consists of the percentage of anterior VB compression with respect to the average height of the anterior vertebral bodies immediately cephalad and caudad to the injury level (formula: V2/[(V1+V3)/2]*100%) [20] (Fig 1).

Kyphotic deformity was defined as negative [16], and lordotic deformity as positive (+).

Clinical outcomes were evaluated using a visual analog scale (VAS) for pain.

### Follow-up criteria

FU visits were scheduled 3 months post-OP as well as prior to implant removal at least 9 months after index surgery. Analysis of radiographic and CT images was performed pre- and post-OP, at 3 months after the index surgery and prior to implant removal. Due to the acute nature of the injury the radiological assessment (radiographs as well as CT-images) at the day of the injury as well as post-OP was usually performed in prone position. At 3 months post-OP as well as at FU prior to implant removal the patients were fully mobile. Accordingly, conventional radiographs were taken having the patient in a standing position.

Of note, the measurement in 2 different positions of the patient (prone at the time of injury/post-OP vs. standing at 3 months FU/FU prior to implant removal) represents a potential confounder. Regarding the radiographic evaluation we were confident that the effect of positioning (prone vs. standing) should be subtle expecting the fracture to be healed at 3 months FU and FU prior to implant removal.

### Statistical analysis

Statistical analysis was performed using SPSS 25.0 software (SPSS Inc, Chicago, IL). Comparisons between independent groups of continuous variables were performed by nonparametric Mann–Whitney U-test. For comparison of radiographic findings between different time point’s non-parametric Wilcoxon-test for paired samples was used. The statistical significance level was set to P≤0.05.

## Results

There were no significant differences between the two groups with regard to their preoperative indices (Table 3).

**Table 3:** Patient characteristics.

		Control Group N = 12	Study Group N = 30
**Sex, number (%)**			
	Males	6 (50)	22 (73)
	Females	6 (50)	8 (27)
Age, years*		44.6 ±15	52.9 ± 11.1
Body mass index*		18.94 ± 3.3	21.3 ± 5.8
**Injury mechanism (number (%))**			
<Fall from (number (%))	Low height (<1m)	4 (33.3)	16 (53.3)
	High height (>1m)	5 (41.6)	5 (16.7)
Road Traffic Crash		1 (8.4)	5 (16.7)
Other		2 (16.7)	4 (13.3)
**FU prior to implant removal (months)**		23.2 ± 14.4	39.9 ±33.1

*Mean plus/minus SD.

The mean preoperative local KA, measured at the level of the fracture, was 12.6° in the study group and 10° in the control group, which was not found to be of significant difference.

**Fig 2:**
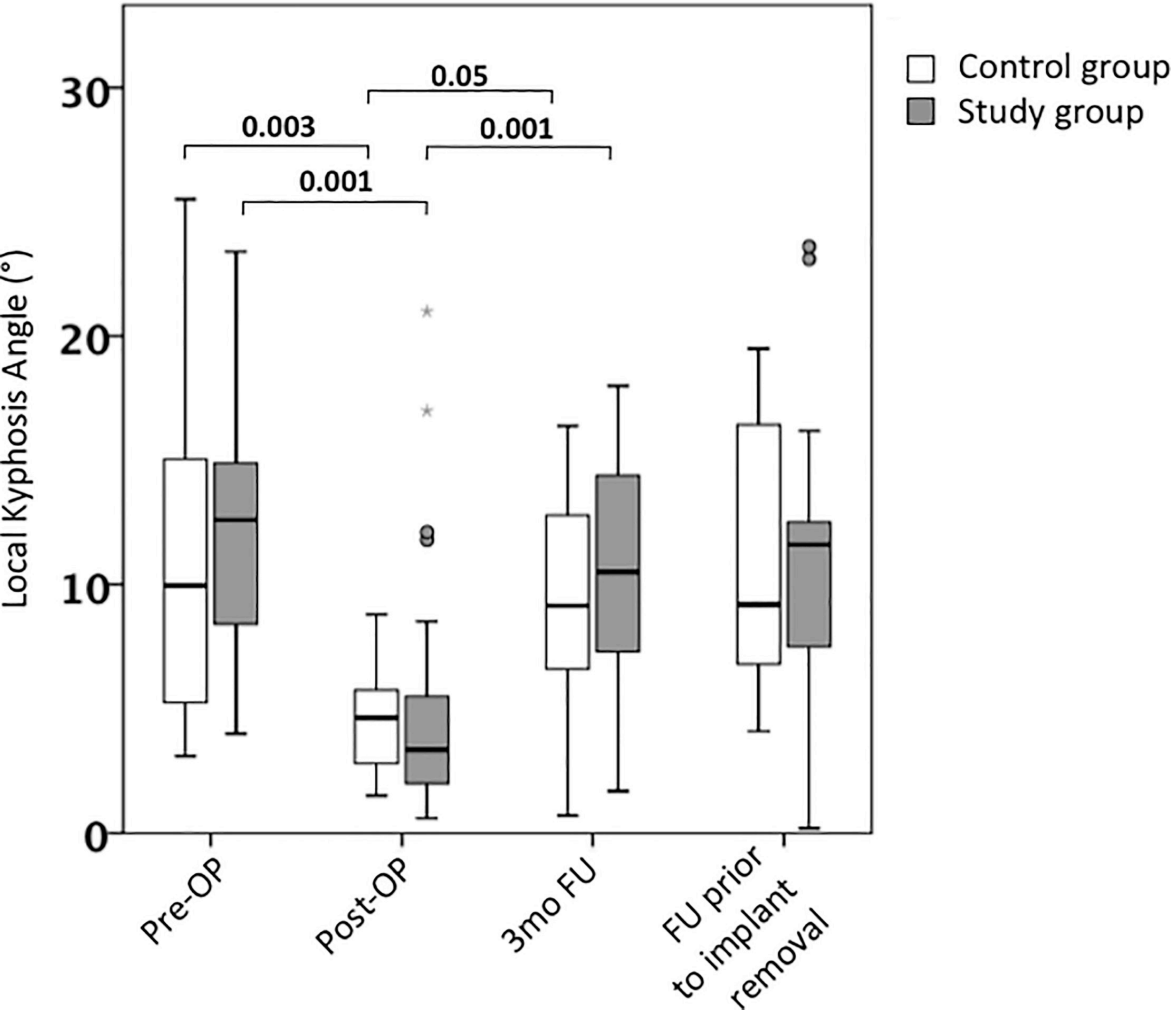
Local kyphosis angle. Comparison of local kyphosis angle (Cobb) between patients who received posterior instrumentation only (control group) and patients who received balloon kyphoplasty of the fractured vertebrae in addition to posterior instrumentation (study group). Values are reflected as mean and standard error of the mean.

Mean local KA was significantly corrected in both groups (3,4° in the study group (P = 0.001) and 4,6° in the control group (P = 0.003) respectively). A striking loss of correction was observed post-OP: local KA decreased to 10.5° in the study group (P>0.005) (Fig 2).

Similarly, the control group sustained a decrease of local KA to baseline level, which was found to be significant (P = 0.05). Details are shown in Table 4.

**Table 4:** Results of radiographic measurements.

	Local KA (°)		Segmental KA (°)		VBCR		AVBC %	
	Control Group	Study Group	Control Group	Study Group	Control Group	Study Group	Control Group	Study Group
**Pre-OP**	9.95±6.59	12.6±4,81	9±8.1	6.85±5.76	0.82±0.2	0.77±0.17	75.6±19.13	71.94±12.3
**Post-OP**	4.65±2.15	3.35±4.79	7.45±6	6.4±5.84	0.93±0.14	0.97±0.12	92.13±9.7	94.78±19.95
**3mo FU**	9.15±4.91	10.5±5	7.5±8.44	11.1±6.84	0.83±0.15	0.76±0.13	78.49±17.44	74.71±10.1
**FU prior to implant removal**	9.2.45±6.1	11.6±6.03	11.3±9.2	10.6±8.65	0.8±0.18	0.83±0.14	76.92±18.98	78.17±14.74

Mean ± SD demonstrating the differences in Local Kyphosis Angle (KA), Segmental KA, VBCR (Vertebral body compression rate) and ABVC % (Anterior VB compression percentage) pre-OP, post-OP at 3 months follow-up (FU), and at 1 year FU. P-Values are included in the Figures.

**Fig 3:**
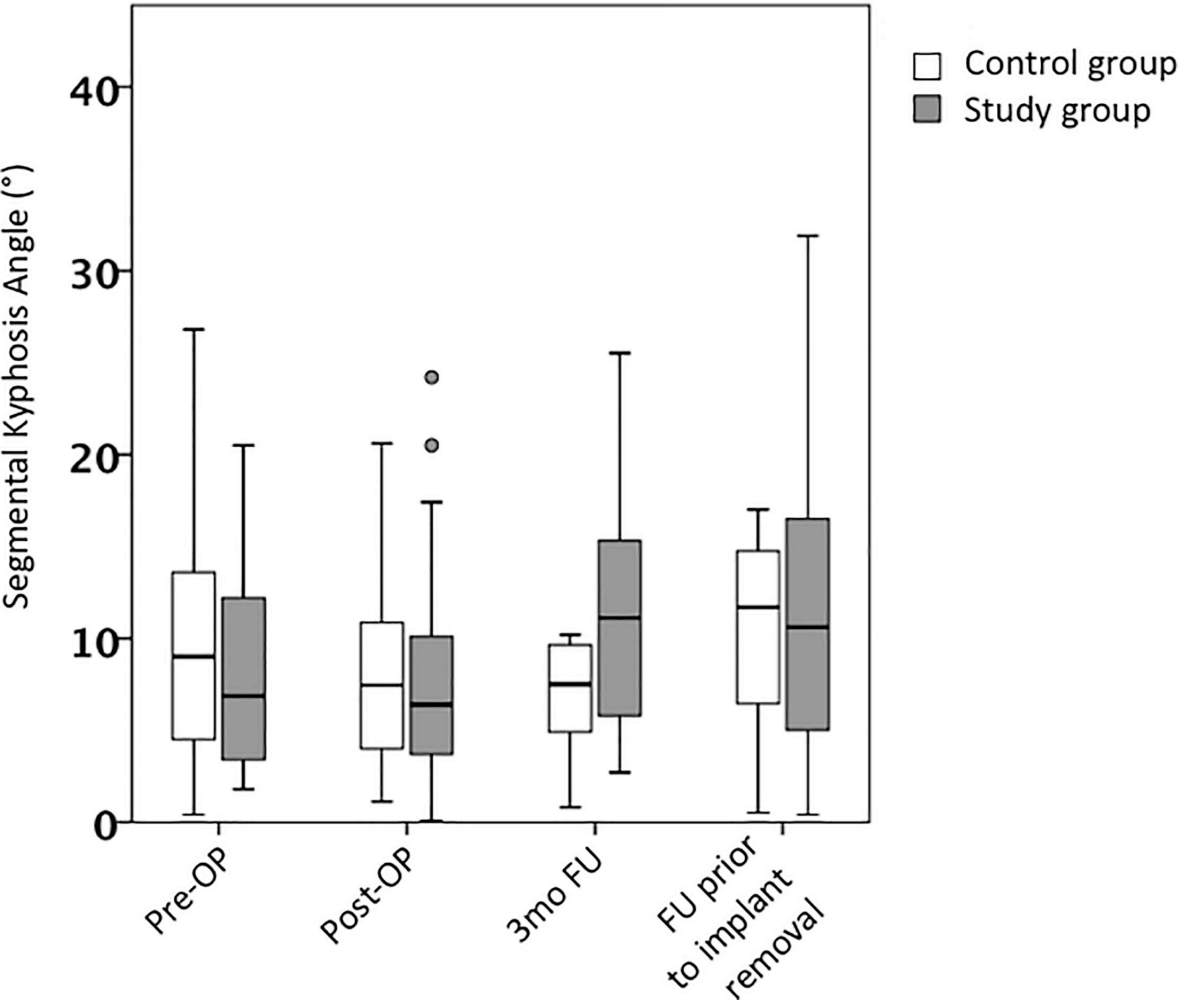
Segmental kyphosis angle. Pre- and postoperative segmental kyphosis angle. Values are reflected as mean and standard error of the mean.

After surgery a subtle improvement of segmental KA was observed from pre-OP 9° to post-Op 7,5° (n.s.) in the control group. We found a significant loss of correction upon final FU. SK decreased to 11.3° at last follow-up in the control group, which was found to be significant (P = 0.028) (Fig 3). Similar observations were made in the study group, although changes were subtler and were not found to be of significance.

**Fig 4:**
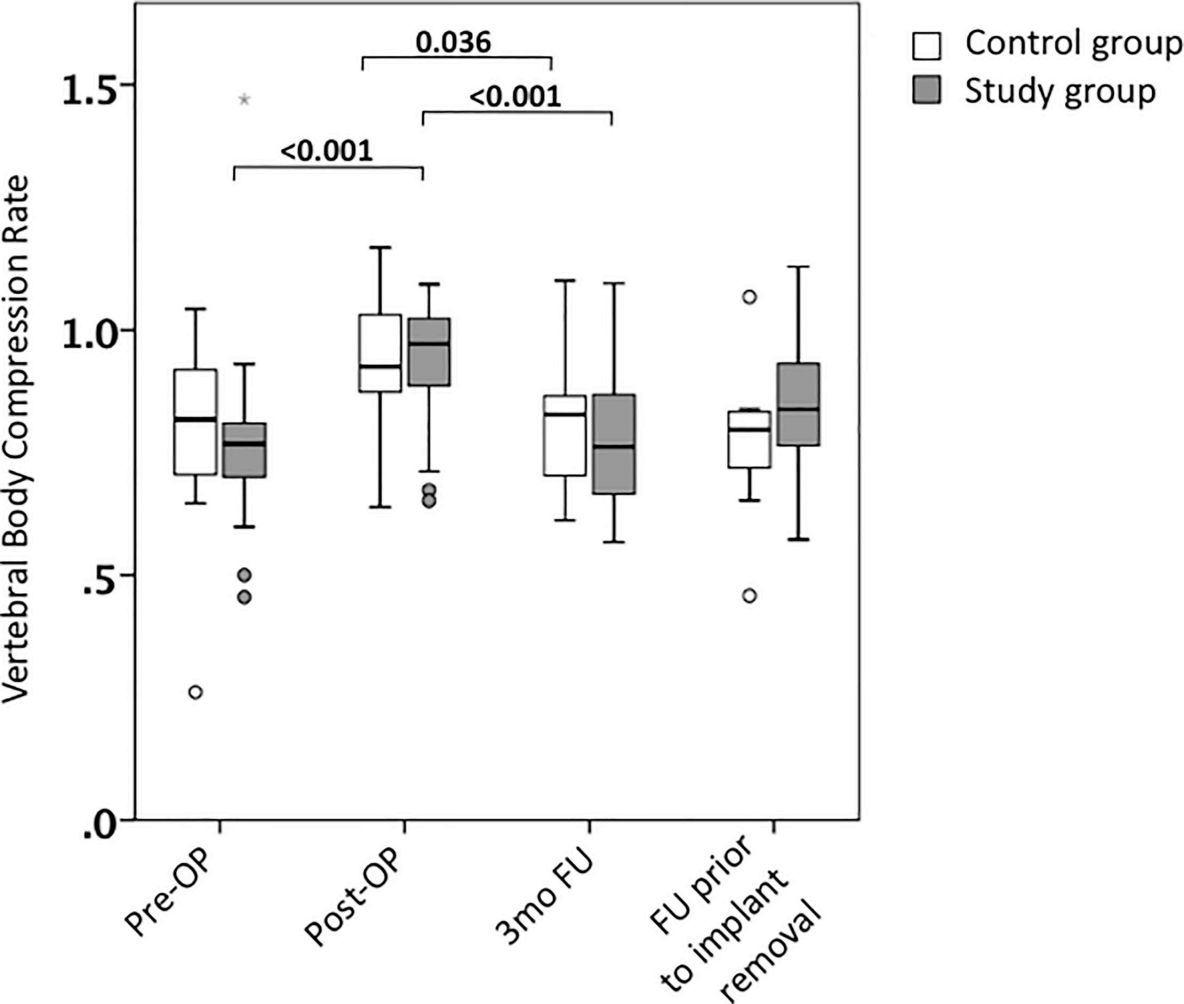
Vertebral body compression rate (VBCR). Pre- and postoperative VBCR in control and study group. Values are reflected as mean and standard error of the mean.

The VBCR significantly improved by additional BK from 0.77° to 0.97°, which was found to be significant (P = 0.001). This is in contrast to postoperative results found in the control group, where reduction was subtler from 0.82° to 0.93° (Fig 4). We identified a significant loss of reduction upon follow-up in both groups.

**Fig 5:**
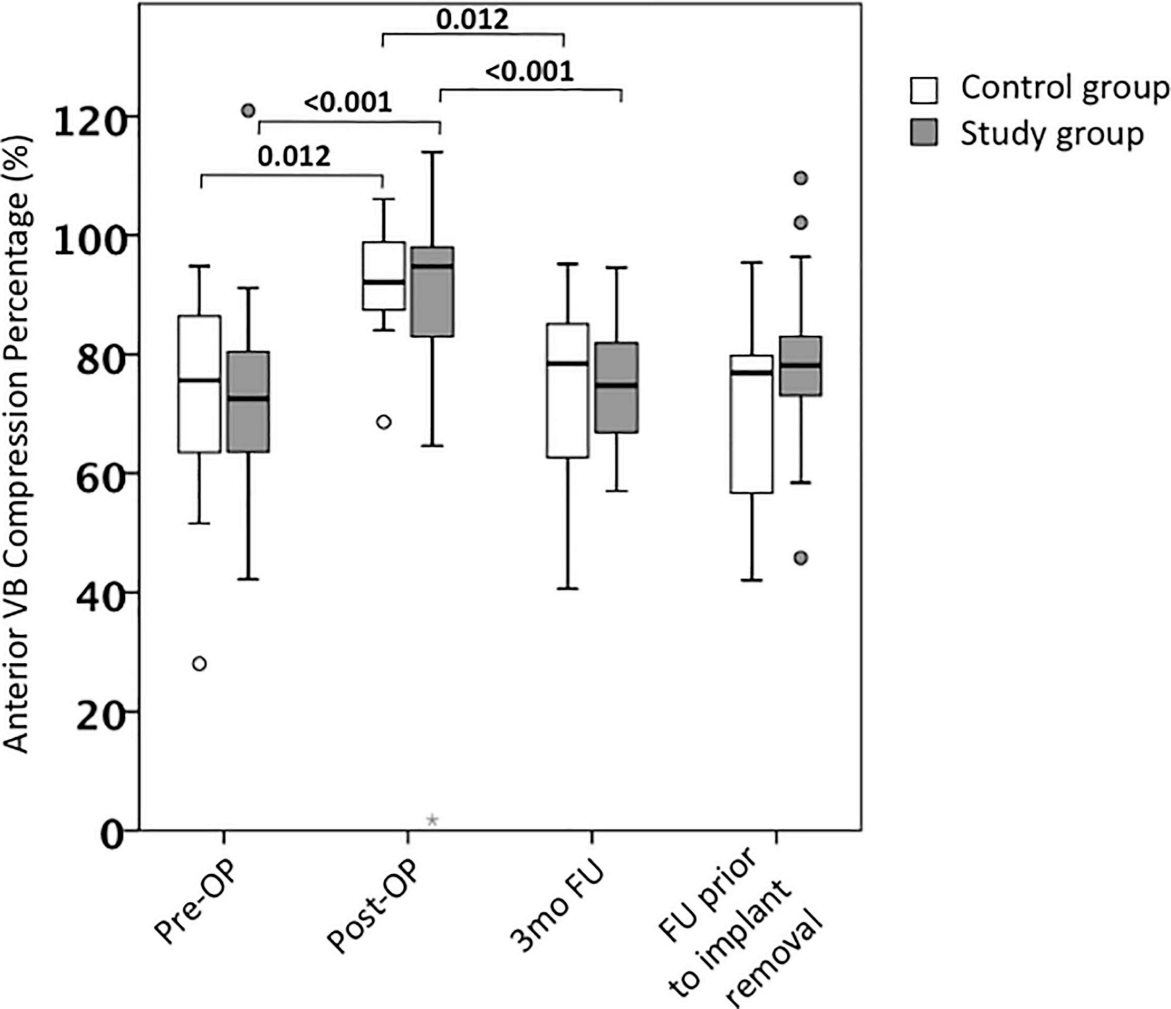
Anterior vertebral body compression percentage. Pre- and postoperative AVBC. Values are reflected as mean and standard error of the mean.

The AVBC revealed a significant endplate reduction in both groups. Within the control group the VB was reduced to approximately 92% of the estimated pre-injury VB height (Fig 5). Results in the study group were even more pronounced: Additional BK resulted in an endplate reduction from approximately 72% pre-OP to almost 95% of the estimated pre-injury VB height. Nevertheless, this striking reduction was not sustained until follow-up, where AVBC converged towards pre-OP measurements.

The patients’ pain levels improved significantly after the operation, since the preoperative VAS averaged 7.2±2.4 and improved to 2.7±1.3 (P<0.001) until FU.

### Complications

Posterior cement leakage was observed in one patient who remained asymptomatic. We observed cement leakage into the vertebral disk space in four patients. There was no worsening of the neurological condition of any of the patients post-OP. In this study we did not observe screw loosening or cut-outs, nor adjacent level fractures that would have made revision surgery necessary. Further, no surgery-related complications, e.g., spine or soft-tissue infections were observed.

None of these patients required a secondary anterior reconstruction.

## Discussion

While results as to the effectiveness of POS alone and stand-alone BK in treating A-type compression fractures of the spine have been controversial, most of the reported studies indicate only limited benefits of the individual procedures [21, 22]. Within this study we aimed to evaluate if an additional BK together with POS using polyaxial screws could retain the intraoperative reduction in a homogenous cohort of 42 young patients (<65a), so as to overcome the limitations of previous reports. While intra-operative reduction was found to be significant in patients with and without BK, we did observe a loss in reduction regardless of the concomitant use of BK in our sample. However, in terms of VAS scale, clinical results appeared to be satisfactory upon implant removal, as most of the patients remained pain-free and no additional surgical intervention was necessary in any of them.

To this end, the combination of both procedures has been repeatedly suggested to potentially improve long term results of A3-type burst fractures [10, 11, 15, 23]. It should be mentioned that these case study series were based on sample sizes of <20 patients without relevant control groups. Despite promising results, these reports do not ultimately provide evidence regarding the significance of concomitant BK.

While bone cement is widely used in trauma surgery, potential complications range from mild, e.g., cement leakage into the disc space to neurological deficits or even death, and should therefore be taken into account [24, 25]. Due to the rarity of serious events, we observed no fatal complications in our cohort. Nevertheless, the significance of cement augmentation in A-type compression fractures has to be critically evaluated in view of potential hazards. Recently, Piazzolla et al. questioned the significance of additional bone cement application for burst fractures. In so doing, the authors reported satisfactory VB height restoration by POS in conjunction with kyphoplasty without applying bone cement, thus avoiding cement-associated risks [14].

Nevertheless, despite excellent intra-operative results, we observed a significant post-operative loss of reduction in both study groups. The impact of screw design, especially polyaxial screws, might be crucial, and has to be taken into account. Polyaxial screws are commonly used in stabilization of thoracolumbar fractures as screw positioning and rod insertion is easy through small incisions. Moreover, polyaxial screws are meant to become truly monoaxial as soon as they are locked. Despite this assumption, a more critical picture is shown by mechanical tests, reporting polyaxial screw failure during dynamic testing due to screw-head slippage before the screw or rod experience plastic bending [26, 27]. This limitation is emphasized in biomechanical testing by Kobusch et al. who report inferiority of polyaxial implants regarding the lowest number of cycles to fail [28].

Accordingly, greater stiffness is probably desirable and increased effort must be made to develop polyaxial screws that are truly monoaxial as soon as they are locked. Nevertheless, there is a thin line between desired plasticity and the concomitant risk of loss of reduction. On the contrary, instrumentation using monoaxial screws produces increased stiffness and tends to fail due to breakage or screw pull-out in biomechanical testing as well as in daily routine [28].

The anchorage of screws and the stability of the posterior instrumentation *per se* depend upon various factors as, for instance, bone density, the insertion depth of the screw, the insertion angle and reinsertion. Besides screw design these factors significantly impact pull-out strength of the pedicle screw [29]. Primary augmentation of pedicle screws is reported to be a feasible method to enhance pull-out strength and reduces the risk of loosening in biomechanical testing [30, 31].

While primary screw augmentation would be truly desirable, several disadvantages of bone cement (e.g., cement embolism, tissue damage due to polymerization temperature <70°C), as mentioned above, hinder us to use cement augmentation in every patient. Therefore, this technique is retained for select patients such as osteoporotic patients, multi-level procedures, and revision surgeries.

In an effort to assess technical alternatives for the augmentation of pedicle screws, which potentially overcome cement-associated risks Wegmann et al. identified the IlluminOss^™^ system [32]. IlluminOss^™^ is a radiolucent monomer already in clinical use for various indications such as fixation of radius fractures. IlluminOss^™^ was tested in an experimental cadaveric setup revealing significantly higher failure loads for augmented pedicle screws compared to native screws. While in vivo data are still missing, it is very likely that pull-out failure or loosening could be reduced by the use of IlluminOss^™^ in spine surgery.

While our study adds to the cumulative evidence on this topic the relatively small sample size as well as the retrospective study design have to be acknowledged as limitations of the study. Moreover, the significance of different fracture patterns seen in A-type fractures should be stressed. This has to be considered when reading our report as most patients presented with A3-type fracture. Due to the over-representation of A3-type fractures our findings might be especially true for this subgroup of patients and further investigations will have to document if our findings also apply to other A-type fractures. Taken together, the most appropriate way to treat Type A compression fractures still remains the subject of controversial discussions. Large, randomized clinical trials will be needed to resolve the still largely contradictory findings of recent studies.

## Supporting information

S1 Data (XLSX)
